# Supplementary material for: Kinetically restrained oxygen reduction to hydrogen peroxide with nearly 100% selectivity
Source: Nat Commun. 2022 May 23;13:2808. doi: 10.1038/s41467-022-30411-7 (PMC9127111; doi:10.1038/s41467-022-30411-7)
Supplement: Supplementary file 1 — Supplementary Information [file 41467_2022_30411_MOESM1_ESM.pdf]

Supplementary Information

Kinetically restrained oxygen reduction to hydrogen peroxide with nearly 100% selectivity

Jinxing Chen,[1,2] Qian Ma,[1,2] Xiliang Zheng,[1] Youxing Fang,[1] Jin Wang,[3*] and Shaojun Dong[1*]

1 State Key Laboratory of Electroanalytical Chemistry, Changchun Institute of Applied Chemistry, Chinese Academy of Sciences, Changchun 130022, China;
2 University of Science and Technology of China, Hefei 230026, China;
3 Department of Chemistry and Physics, Stony Brook University, Stony Brook, NY 11794, USA

Corresponding authors. E-mails: jindwang12@163.com; dongsj@ciac.ac.cn

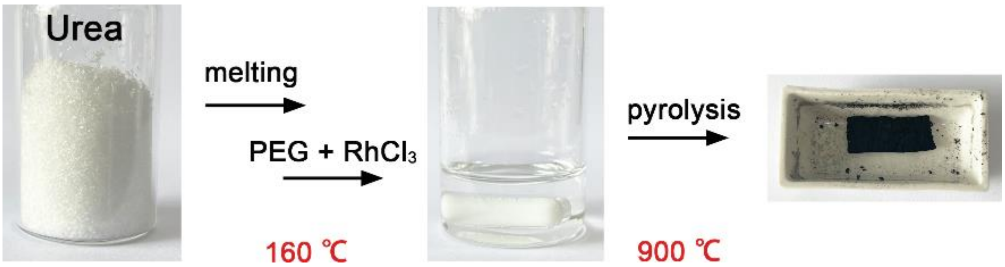

Supplementary Figure 1. The steps of synthesizing Rh_1/NC.

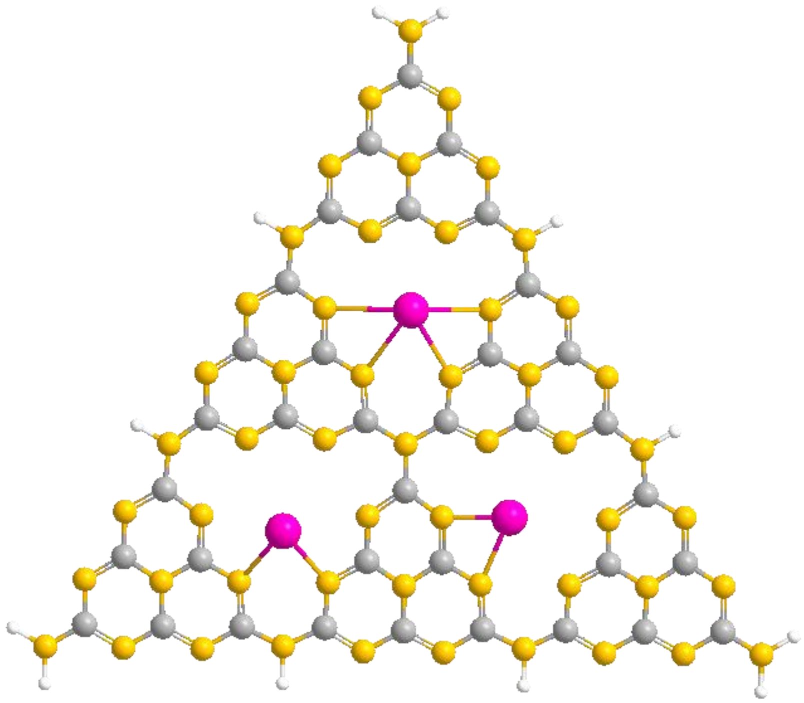

Supplementary Figure 2. The coordination structure of g-C_3N_4 and Rh. Rh, red; N, yellow; C, gray.

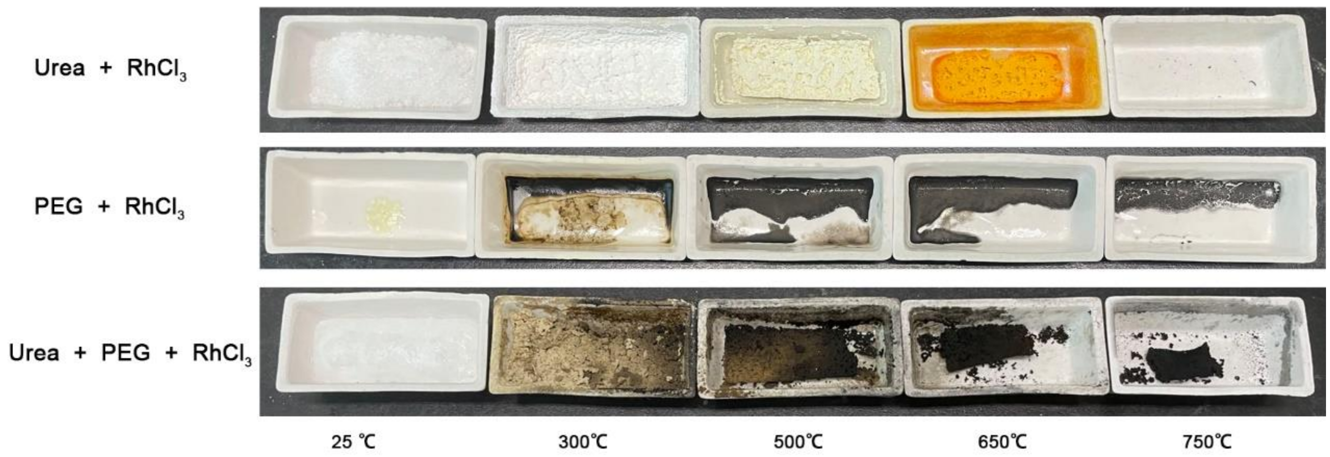

Supplementary Figure 3. Photos of the different mixtures after pyrolysis at different temperatures.

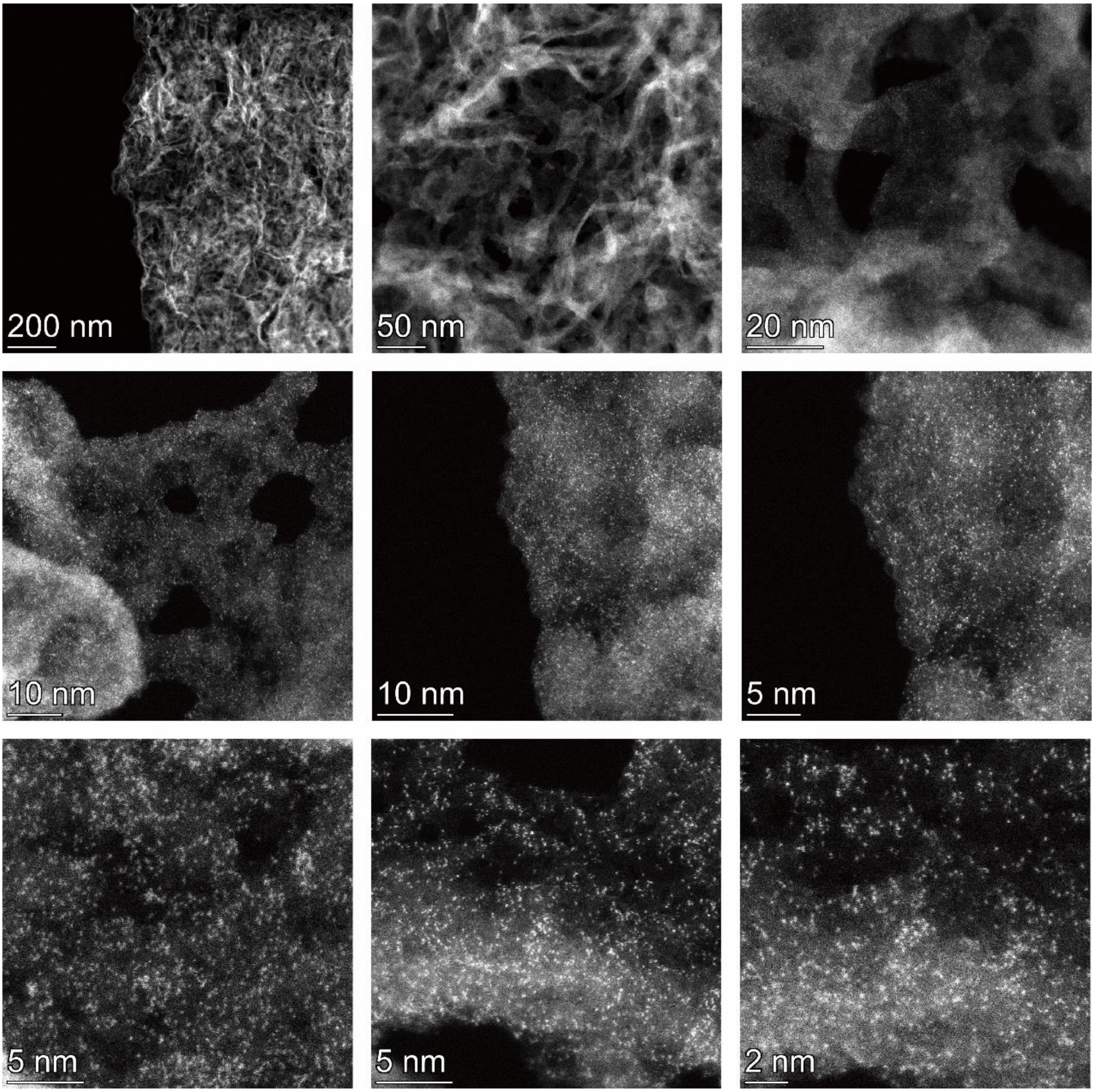

Supplementary Figure 4. HAADF-STEM images of Rh_1/NC with different magnifications. Experiment was repeated three 3 independently with similar results.

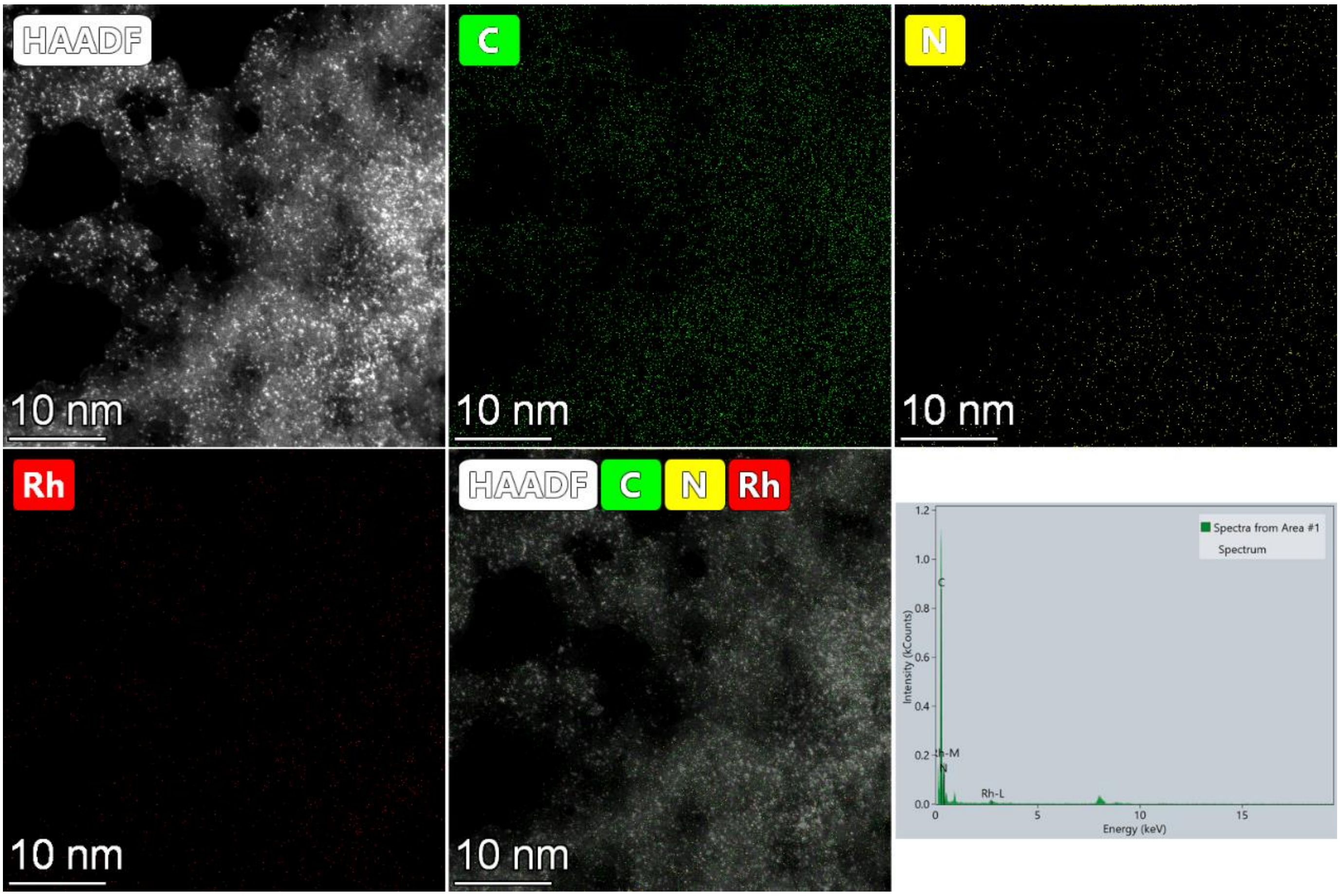

Supplementary Figure 5. HAADF-STEM images and corresponding energy-dispersive X-ray elemental mapping as well as the EDS spectrum of Rh_1/NC. Experiment was repeated three 3 independently with similar results.

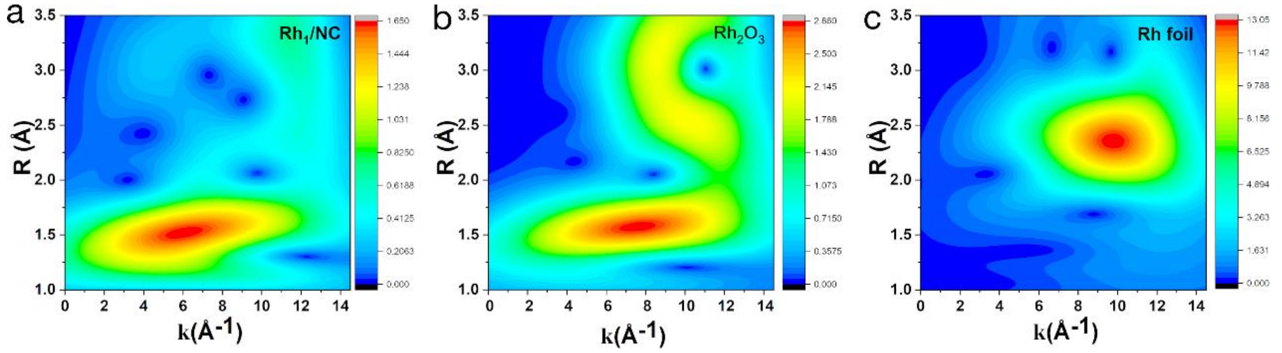

Supplementary Figure 6. Wavelet transforms for the *k*3-weighted EXAFS signals of Rh_1/NC (a), Rh_2O_3 (b), and Rh foil (c).

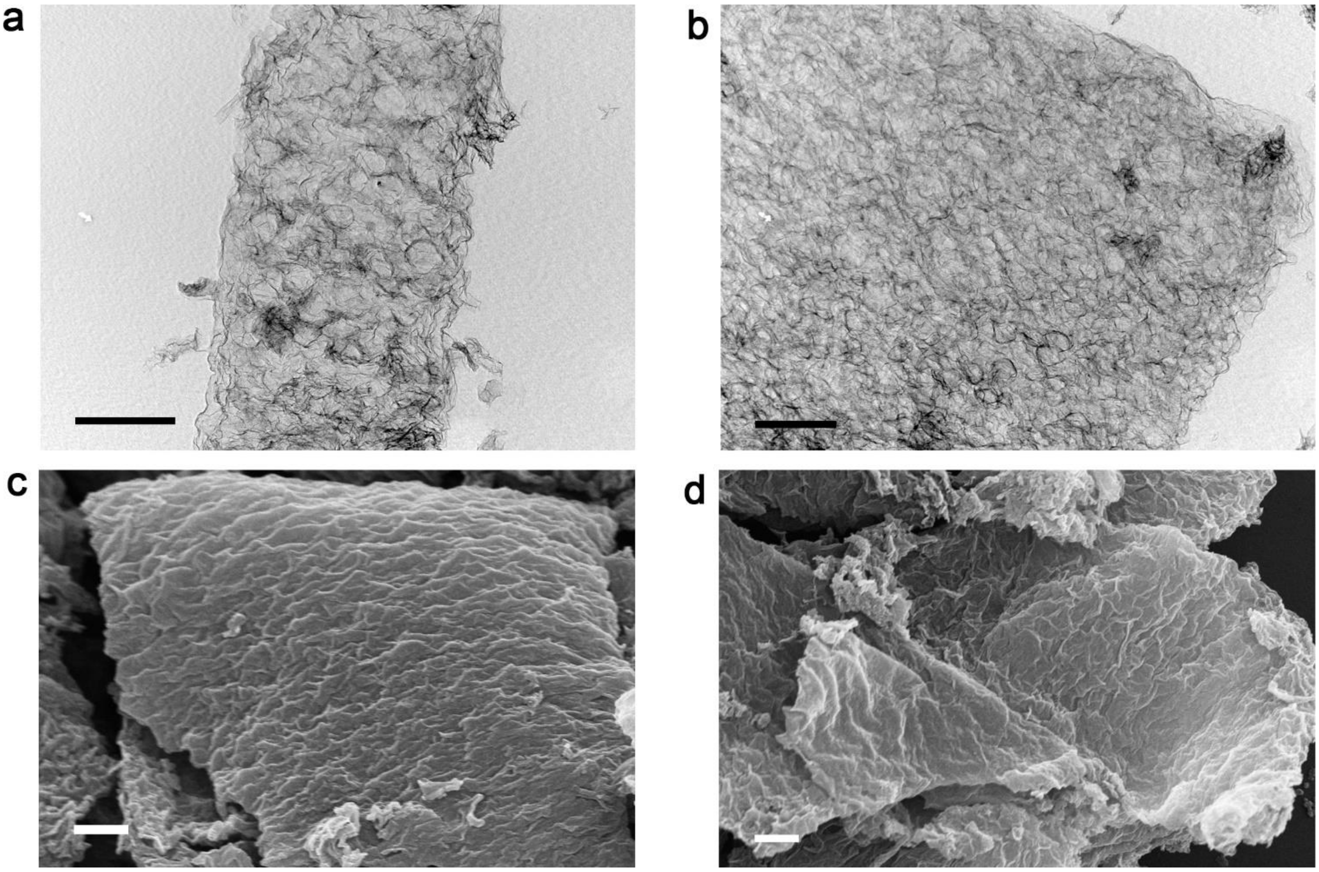

Supplementary Figure 7. TEM images of Ir_1/NC (a) and Co_1/NC (b). SEM images of Ir_1/NC (c) and Co_1/NC (d). Scale bars: 500 nm. Experiment was repeated three 3 independently with similar results.

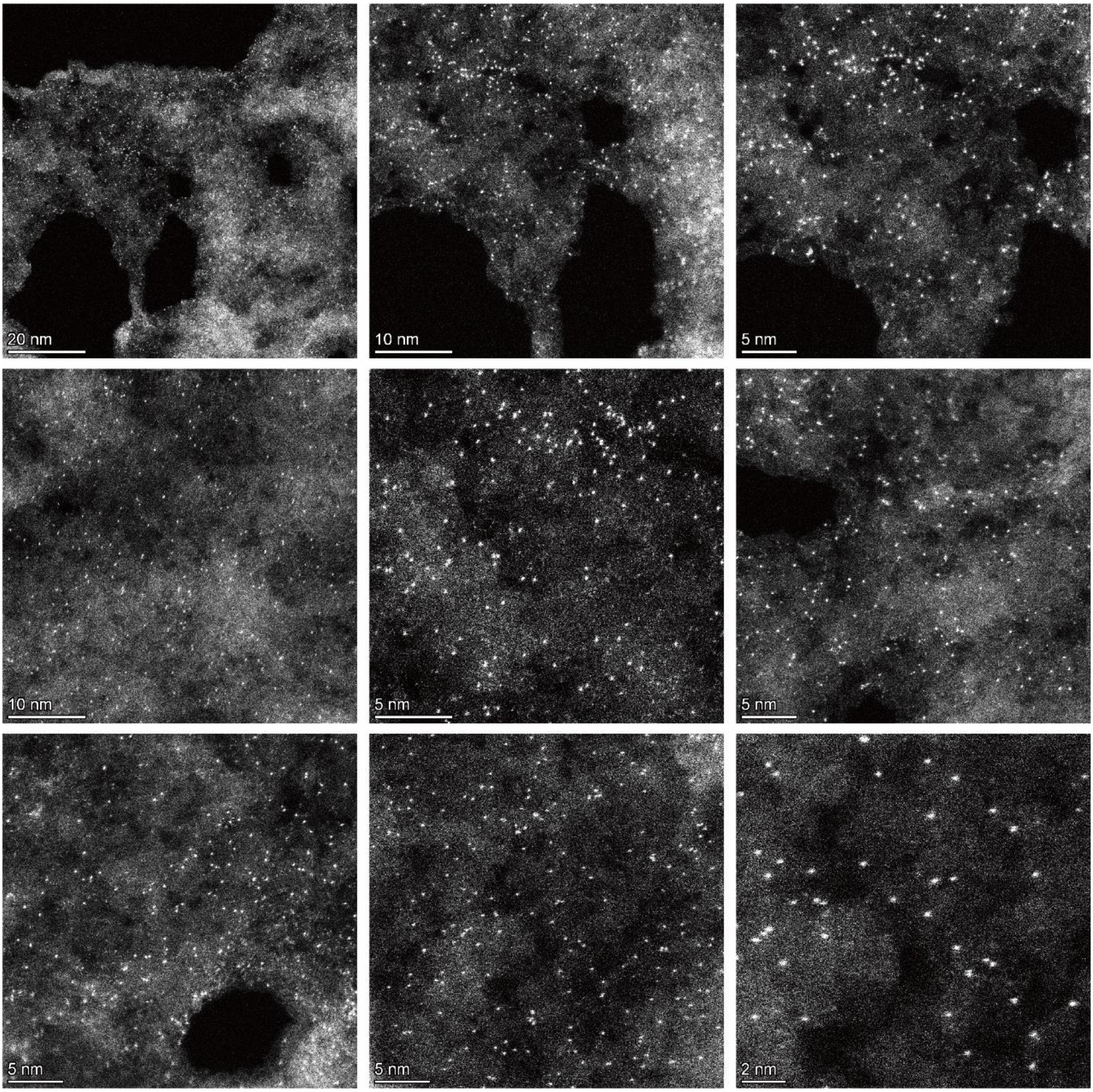

Supplementary Figure 8. HAADF-STEM images of Ir_1/NC with different magnifications. Experiment was repeated three 3 independently with similar results.

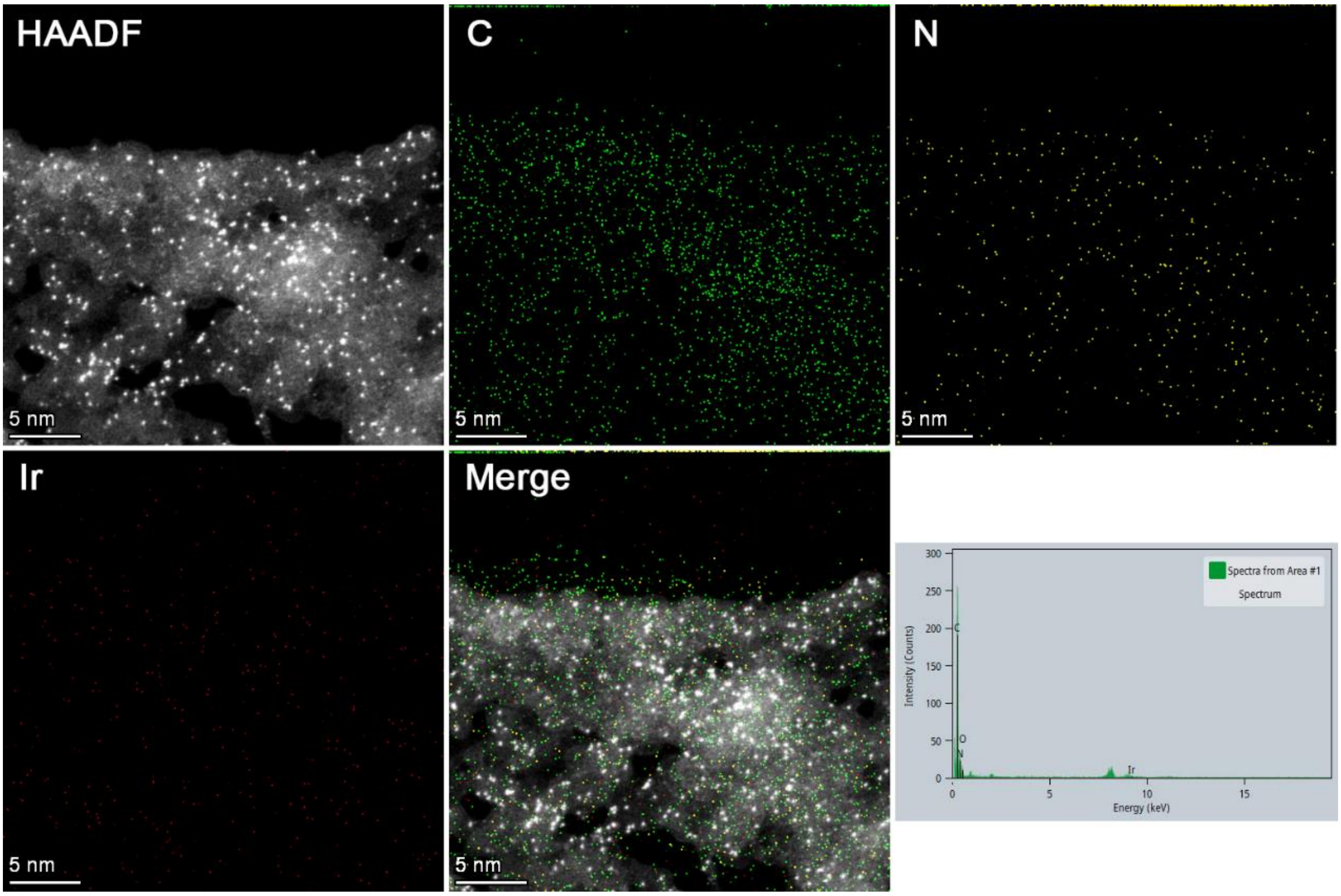

Supplementary Figure 9. HAADF-STEM image and corresponding energy-dispersive X-ray elemental mapping as well as the EDS spectrum of Ir_1/NC. Experiment was repeated three 3 independently with similar results.

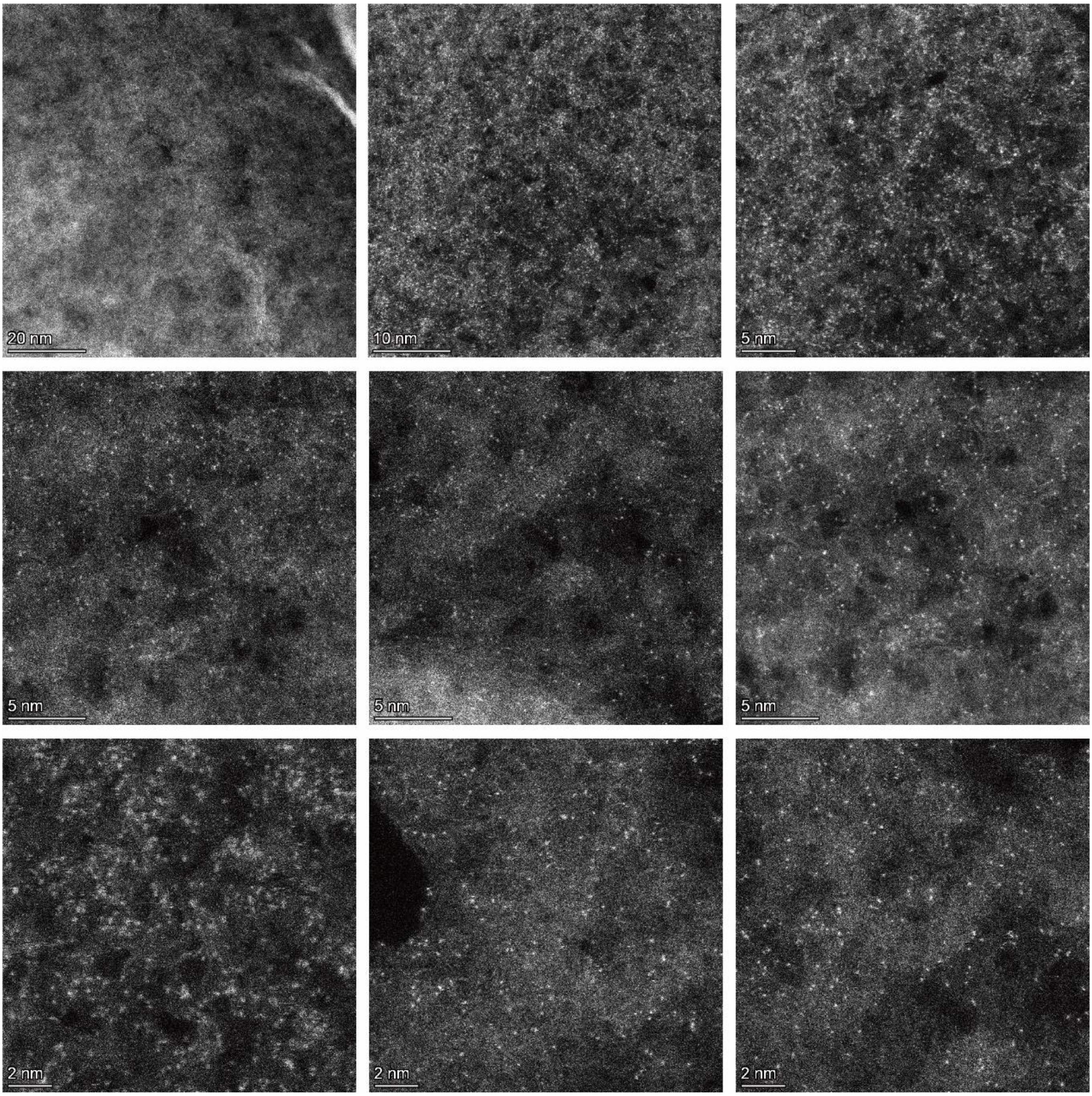

Supplementary Figure 10. HAADF-STEM images of Co_1/NC with different magnifications. Experiment was repeated three 3 independently with similar results.

The relative atomic mass of Co is lower than those of Rh and Ir, so the contrast between the Co atom and carbon substrate in the HAADF-STEM image of Co_1/NC is low compared with those of Rh_1/NC and Ir_1/NC.

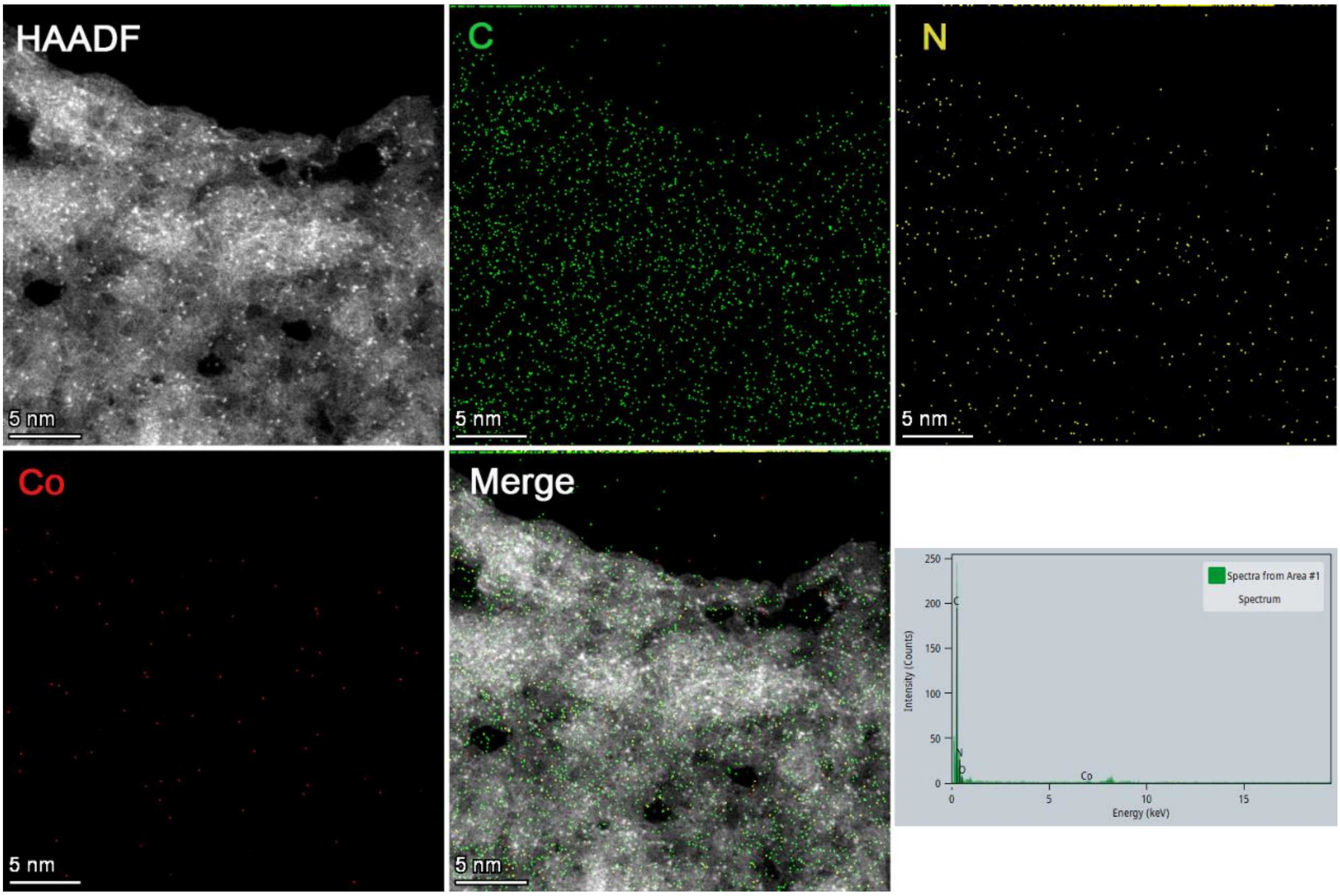

Supplementary Figure 11. HAADF-STEM image and corresponding energy-dispersive X-ray elemental mapping as well as the EDS spectrum of Co_1/NC. Experiment was repeated three 3 independently with similar results.

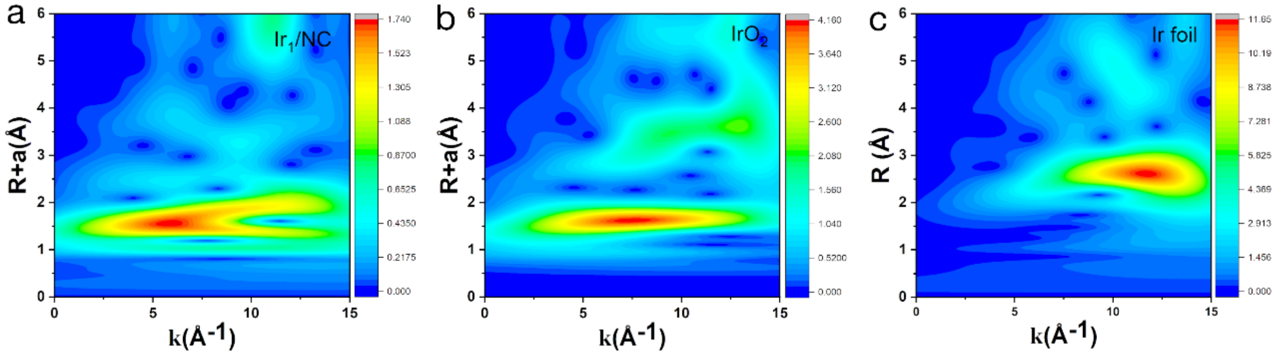

Supplementary Figure 12. Wavelet transforms for the *k*3-weighted EXAFS signals of Ir_1/NC (a), IrO_2 (b), and Ir foil (c).

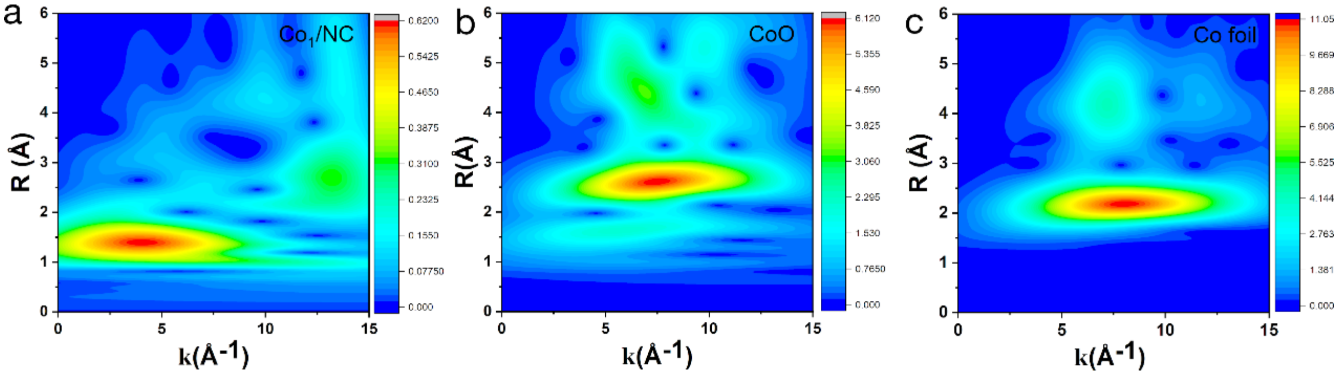

Supplementary Figure 13. Wavelet transforms for the *k*3-weighted EXAFS signals of Co_1/NC (a), CoO (b), and Co foil (c).

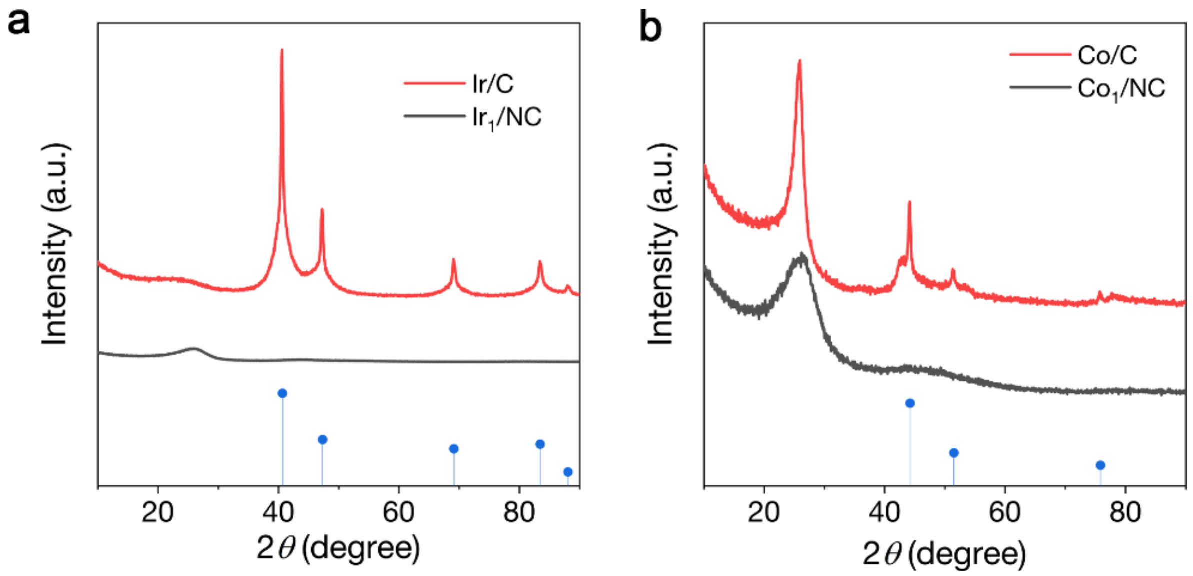

Supplementary Figure 14. (a) XRD patterns of Ir_1/NC and Ir/C. (b) XRD patterns of Co_1/NC and Co/C.

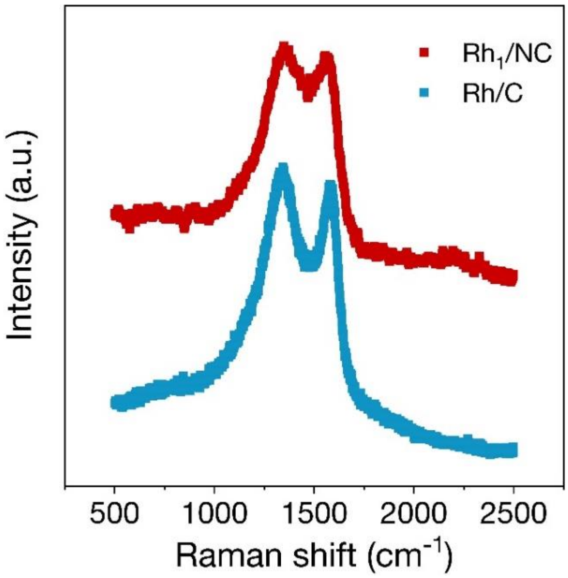

Supplementary Figure 15. Raman spectra of Rh_1/NC and Rh/C.

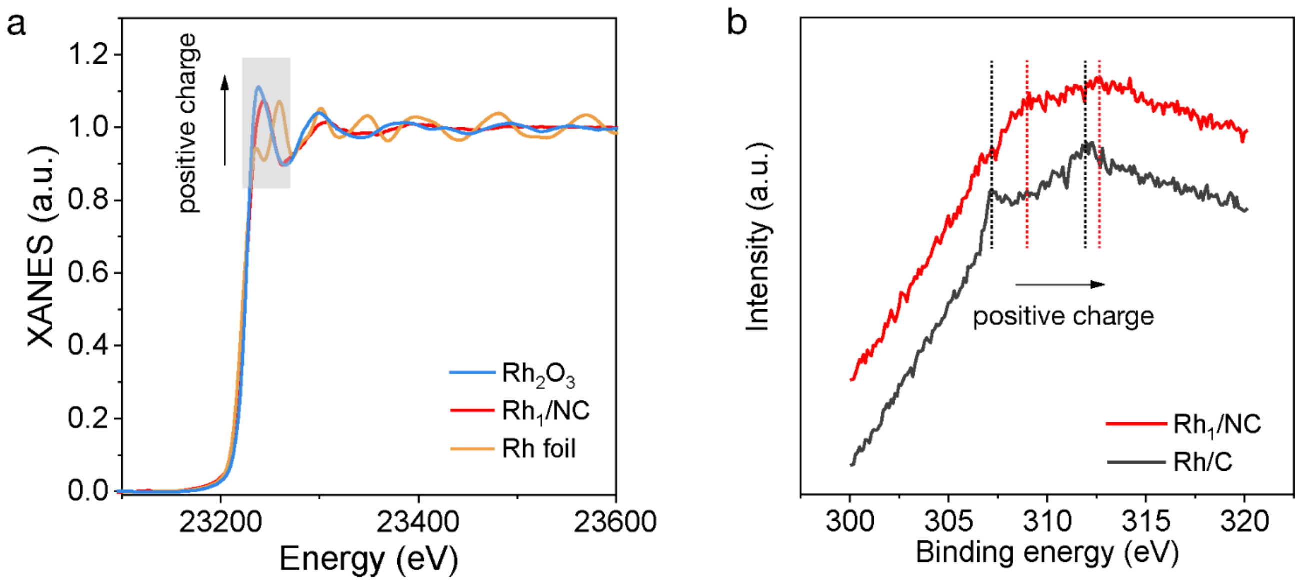

Supplementary Figure 16. (a) Rh K-edge XANES spectra of Rh $_1$/NC, Rh foil, and Rh_2O_3. (b) Rh 3d XPS spectra of Rh $_1$/NC and Rh/C.

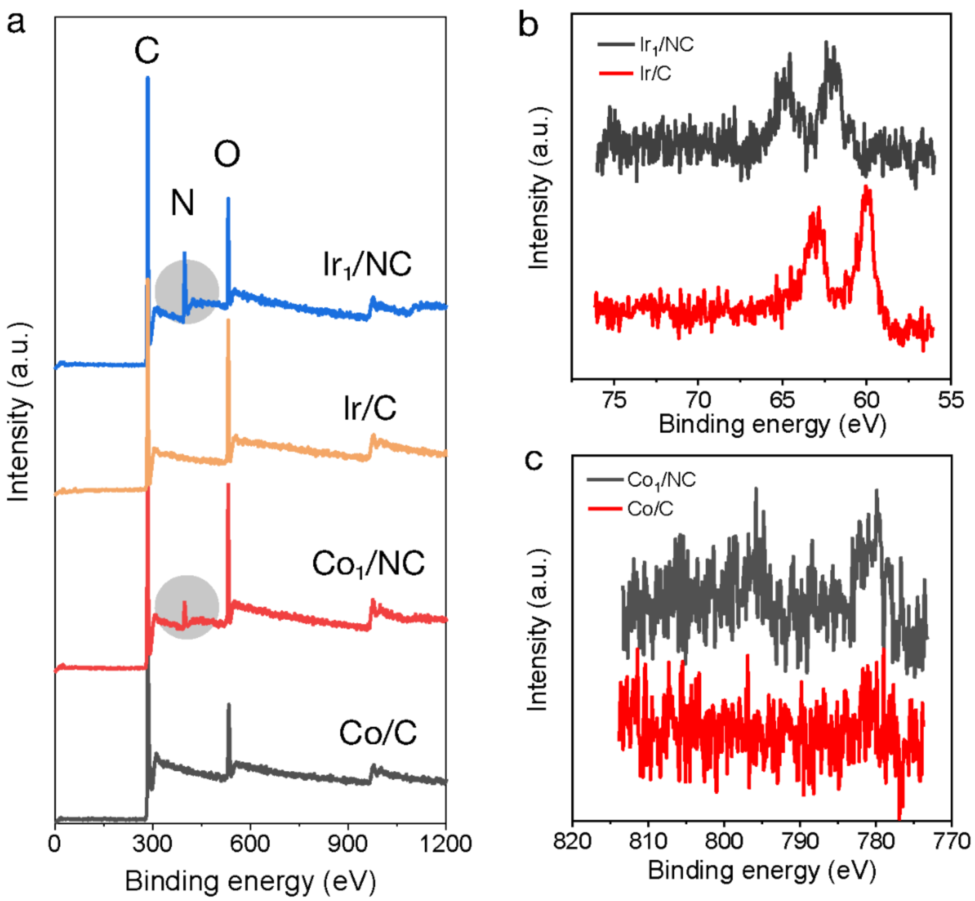

Supplementary Figure 17. (a) XPS survey spectra of Ir_1/NC, Ir/C, Co_1/NC, and Co/C. (b) Ir 4f XPS spectra of Ir_1/NC and Ir/C. (c) Co 2p XPS spectra of Co_1/NC and Co/C.

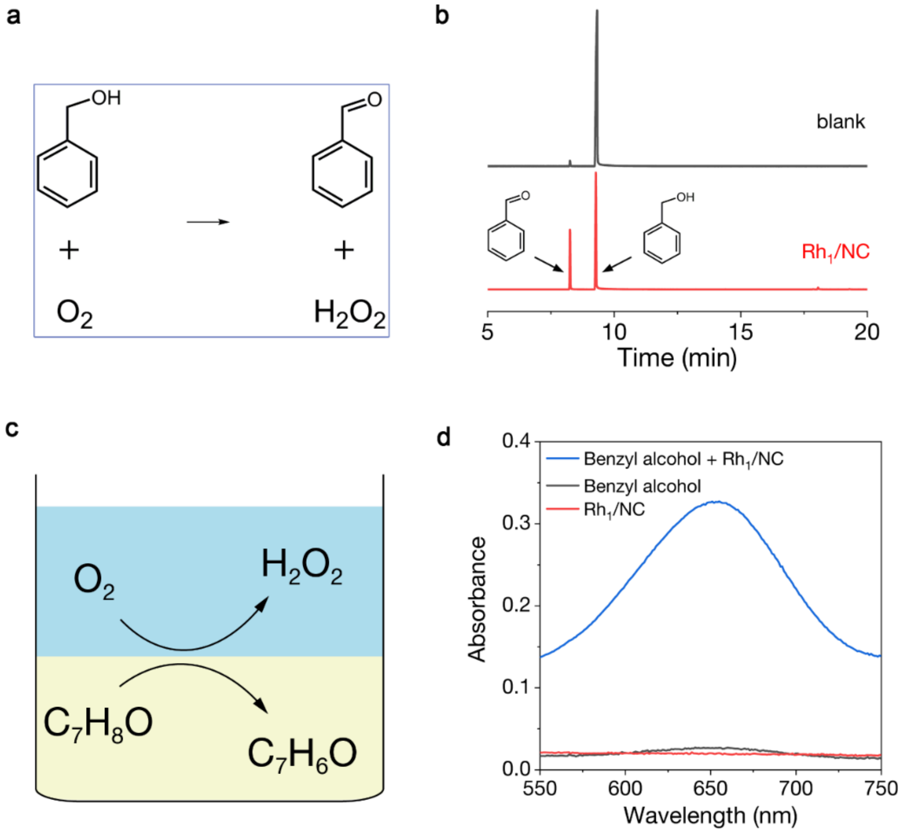

Supplementary Figure 18. (a) Benzyl alcohol oxidation produces H_2O_2. (b) Chromatogram of benzyl alcohol after oxidation in the absence or presence of Rh_1/NC. (c) Schematic illustration of Rh_1/NC-catalyzed benzyl alcohol oxidation and O_2 reduction. (d) UV–vis absorption spectra of different mixture solutions after the addition of HRP and TMB.

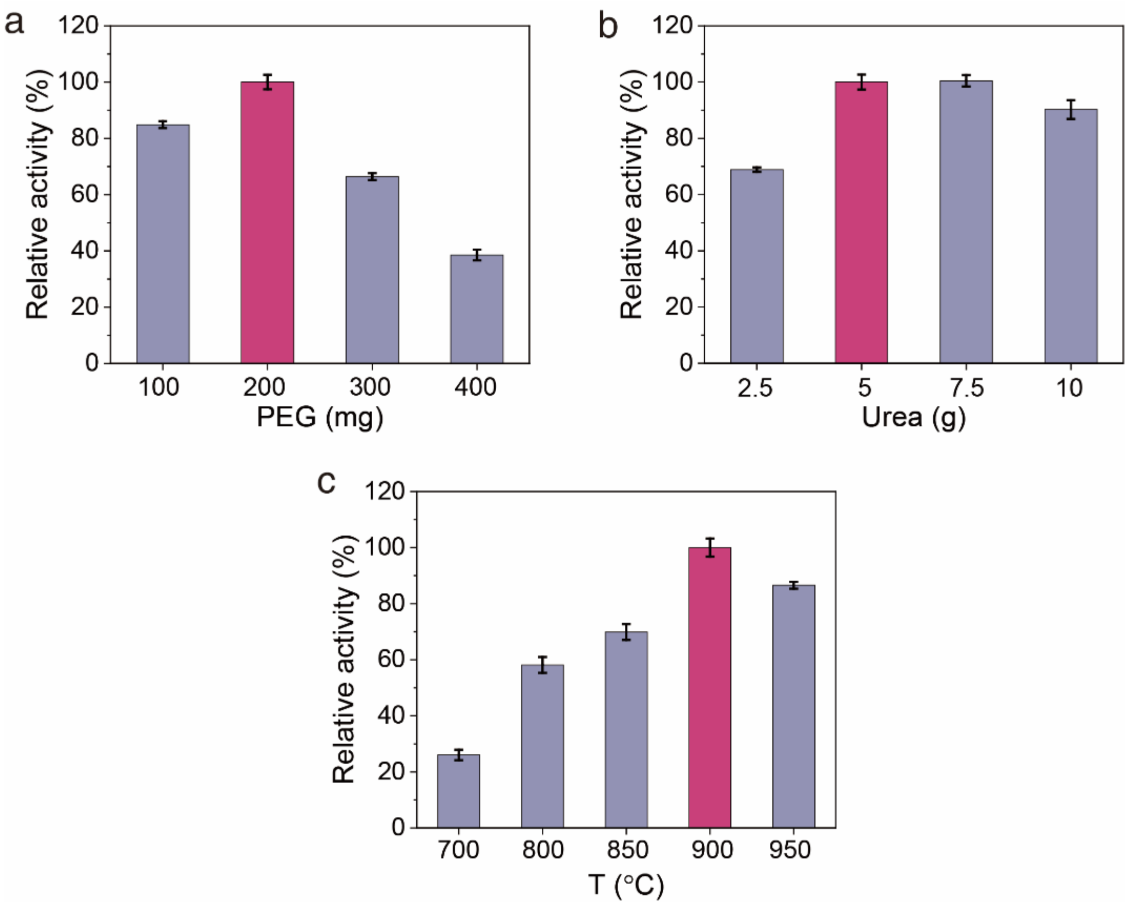

Supplementary Figure 19. Activity (aerobic oxidation of benzyl alcohol) comparison of catalysts obtained under different synthesis conditions. (a) Urea, 5 g; $RhCl_3$, 0.005 mmol; 900 °C. (b) PEG, 200 mg; $RhCl_3$, 0.005 mmol; 900 °C. (c) Urea, 5 g; $RhCl_3$, 0.005 mmol; PEG, 200 mg. Data are presented as mean values (SD). (The error bar represents the standard deviation of 3 independent measurements).

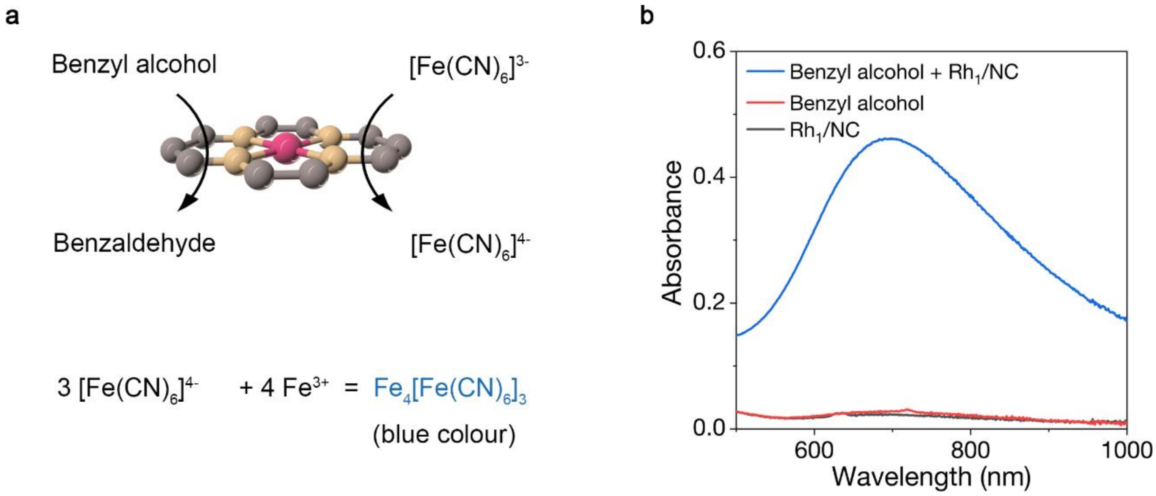

Supplementary Figure 20. (a) Schematic illustration of Rh_1/NC-catalyzed benzyl alcohol oxidation and $[Fe(CN)_6]^{3-}$ reduction. (a) UV–vis absorption spectra of different mixture solutions after the addition of Fe^{3+}.

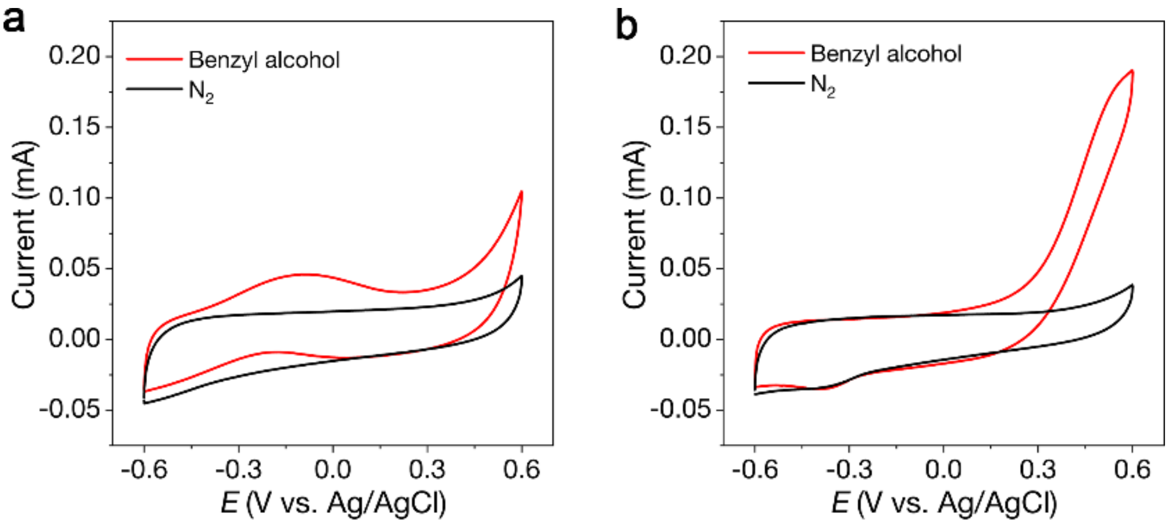

Supplementary Figure 21. CV curves of Ir_1/NC (a) and Co_1/NC (b) catalyzed benzyl alcohol oxidation of 0.1 M NaOH solutions with a scan rate of 100 mV s^{-1}.

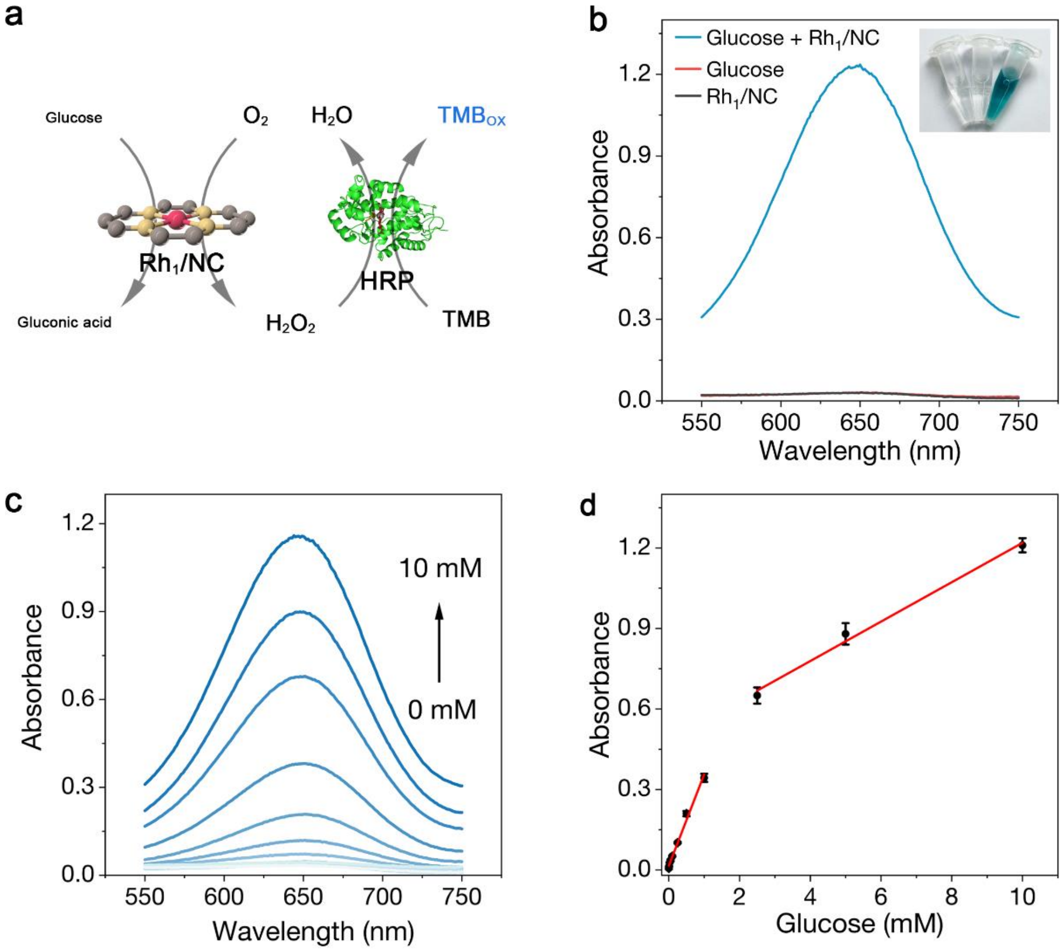

Supplementary Figure 22. (a) Schematic illustration of glucose detection by the typical colorimetric assay with Rh_1/NC as a GOD mimic and HRP. (b) UV–vis absorption spectra of different mixture solutions after the addition of HRP and TMB. (c) UV–vis absorption spectra of the mixture of Rh_1/NC and different concentrations of glucose after the addition of HRP and TMB. (d) Linear plot of absorbance intensity (650 nm) versus glucose concentration. All experiments were conducted in PBS (50 mM, pH=7.4). Data are presented as mean values (SD). (The error bar represents the standard deviation of 3 independent measurements).

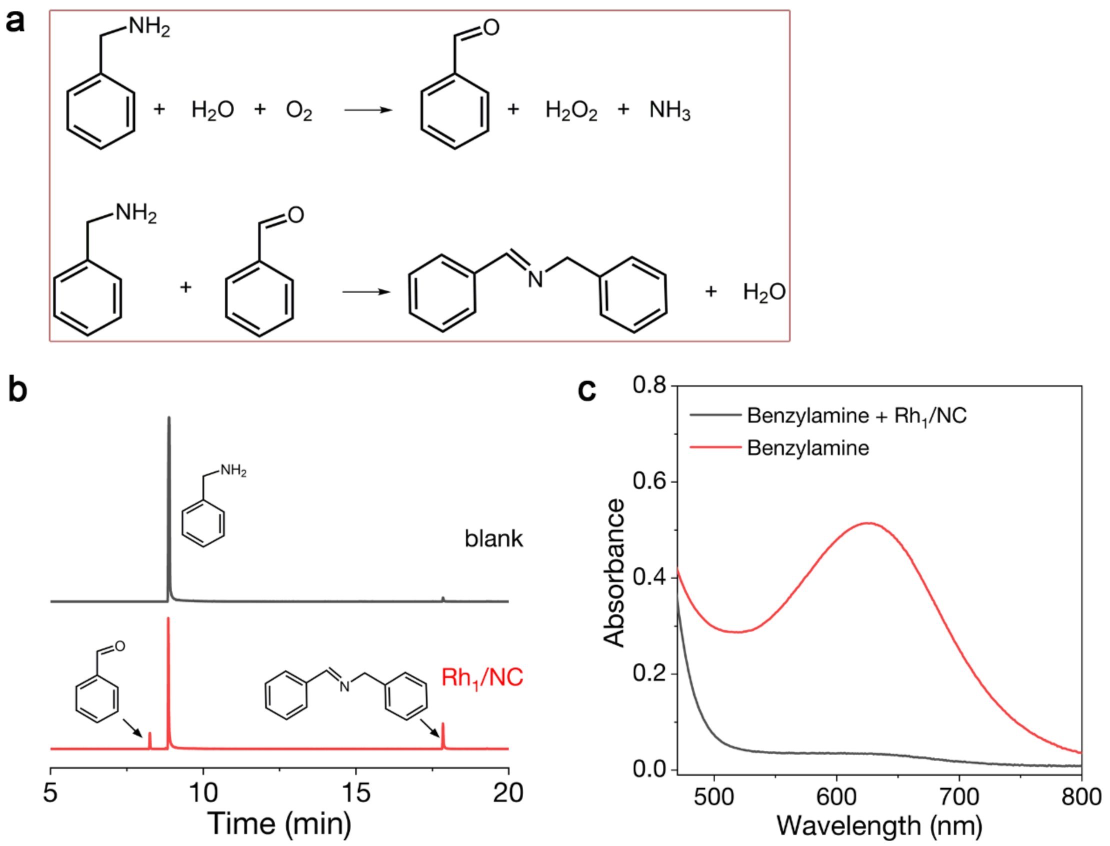

Supplementary Figure 23. (a) Schematic illustration of Rh_1/NC-catalyzed benzylamine oxidation. (b) Chromatogram of benzylamine after oxidation in the absence or presence of Rh_1/NC. (c) UV–vis absorption spectra of different mixture solutions after the addition of reagent for detecting ammonium.

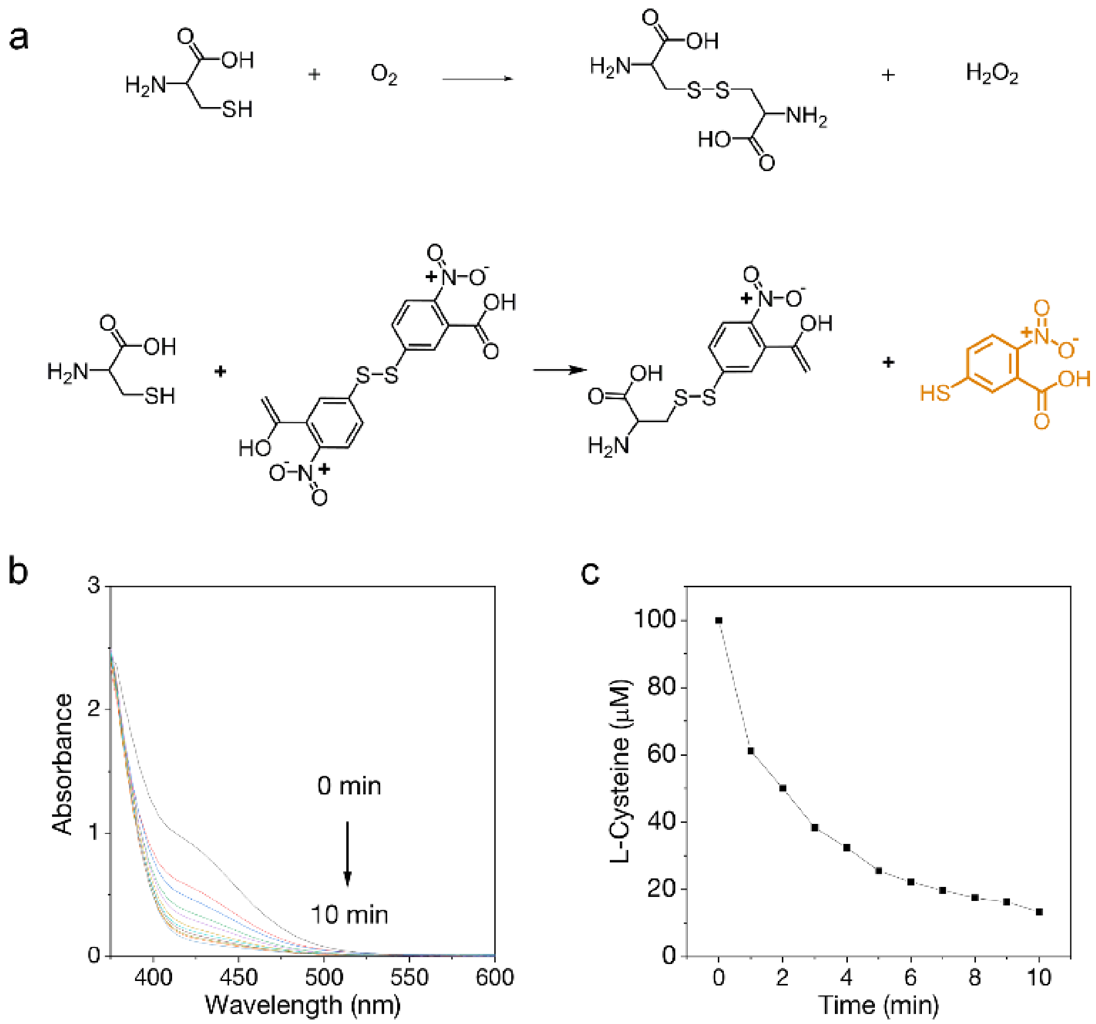

Supplementary Figure 24. (a) Schematic illustration of Rh₁/NC-catalyzed L-cysteine oxidation and colorimetric detection of cysteine by 5,5'-dithiobis-(2-nitrobenzoic acid) (DTNB). (b) Time-dependent absorption spectra change of the reaction mixture containing Rh_1/NC (20 μg mL^{-1}) and L-cysteine (100 μM) after addition of DTNB. (c) L-cysteine concentration change with time in the presence of Rh_1/NC (20 μg mL^{-1}). All experiments were conducted in PBS (50 mM, pH=7.4).

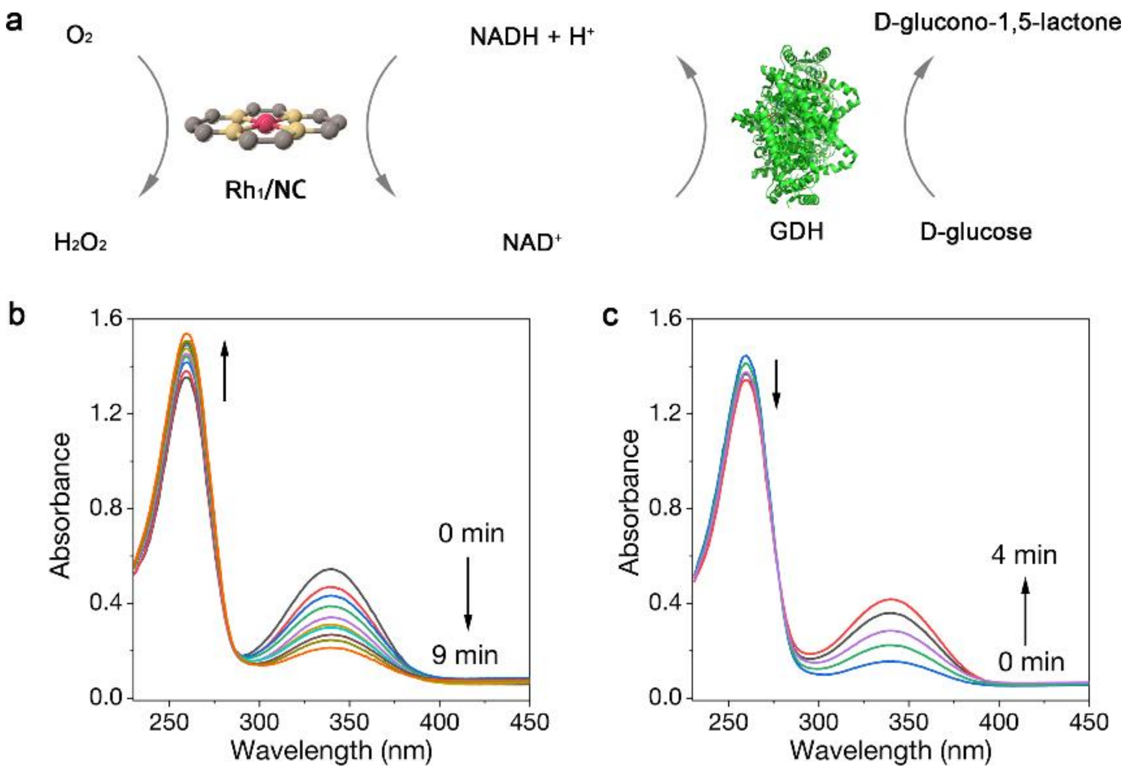

Supplementary Figure 25. (a) Schematic illustration of Rh_1/NC-catalyzed NADH oxidation and NAD^+ reduction catalyzed by glucose dehydrogenase (GDH). (b) Time-dependent absorption spectra change of the reaction mixture containing Rh_1/NC (20 μg mL^{-1}) and NADH (100 μM). (c) Time-dependent absorption spectra change of the reaction mixture in (b) with added GDH and glucose. All experiments were conducted in PBS (50 mM, pH=7.4).

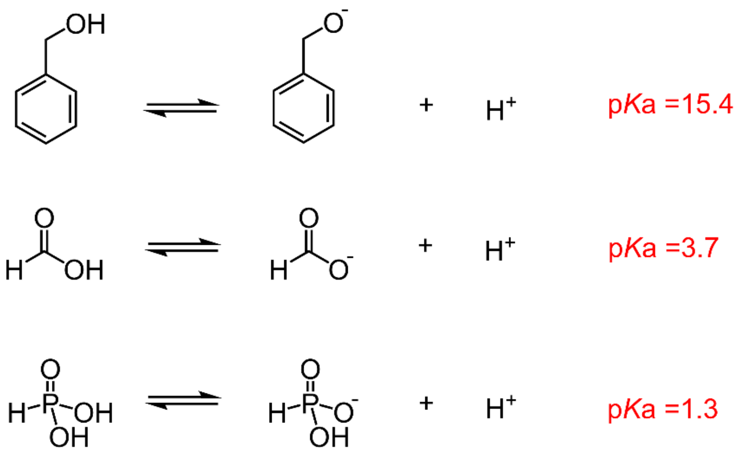

Supplementary Figure 26. The breakage of the O-H bond in the reaction of C_7H_8O, HCOOH and H_3PO_3, individually.

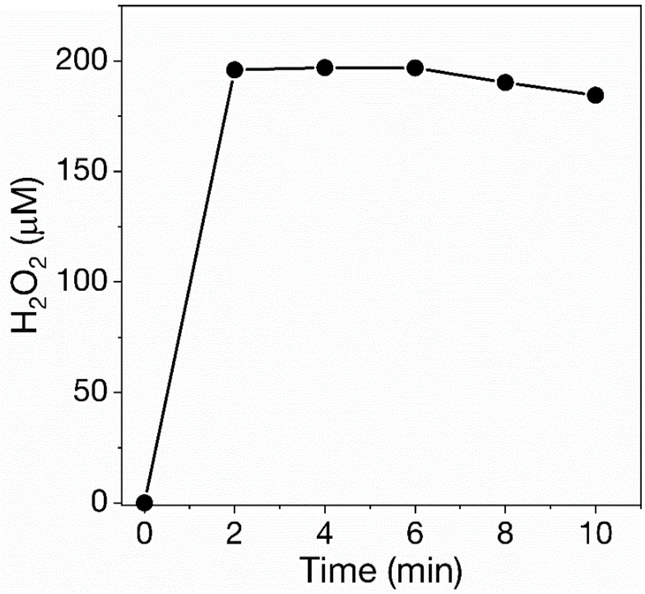

Supplementary Figure 27. Time-dependent H_2O_2 concentration variation in 2.5 mM H_3PO_3+ 2.5 mM KH_2PO_3 in the presence of 50 μg mL^{-1} of Pt/C.

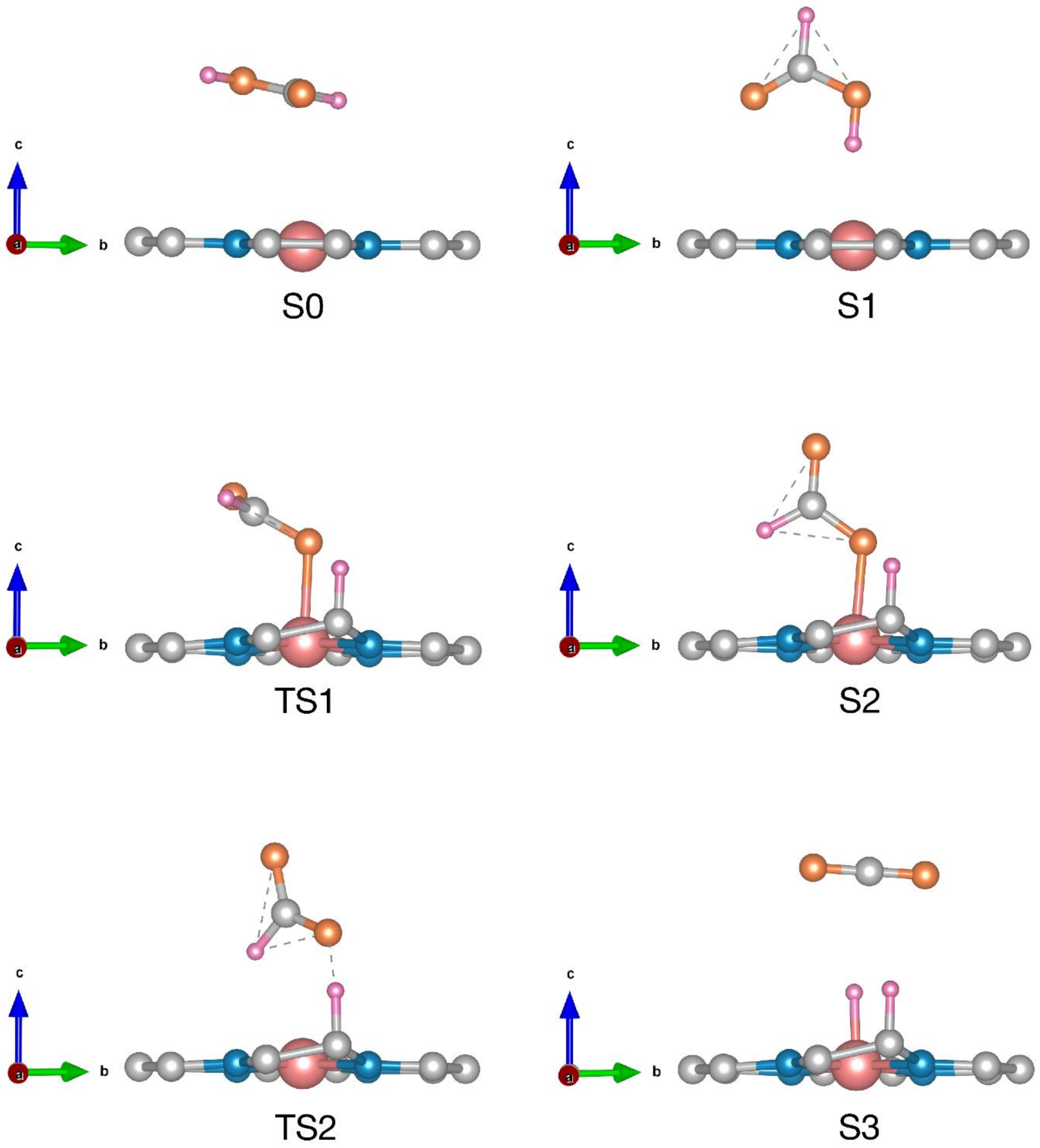

Supplementary Figure 28. Optimized structures of computational models in Rh-catalyzed HCOOH. blue, N; orange, O; gray, C; red, Rh; pink, H.

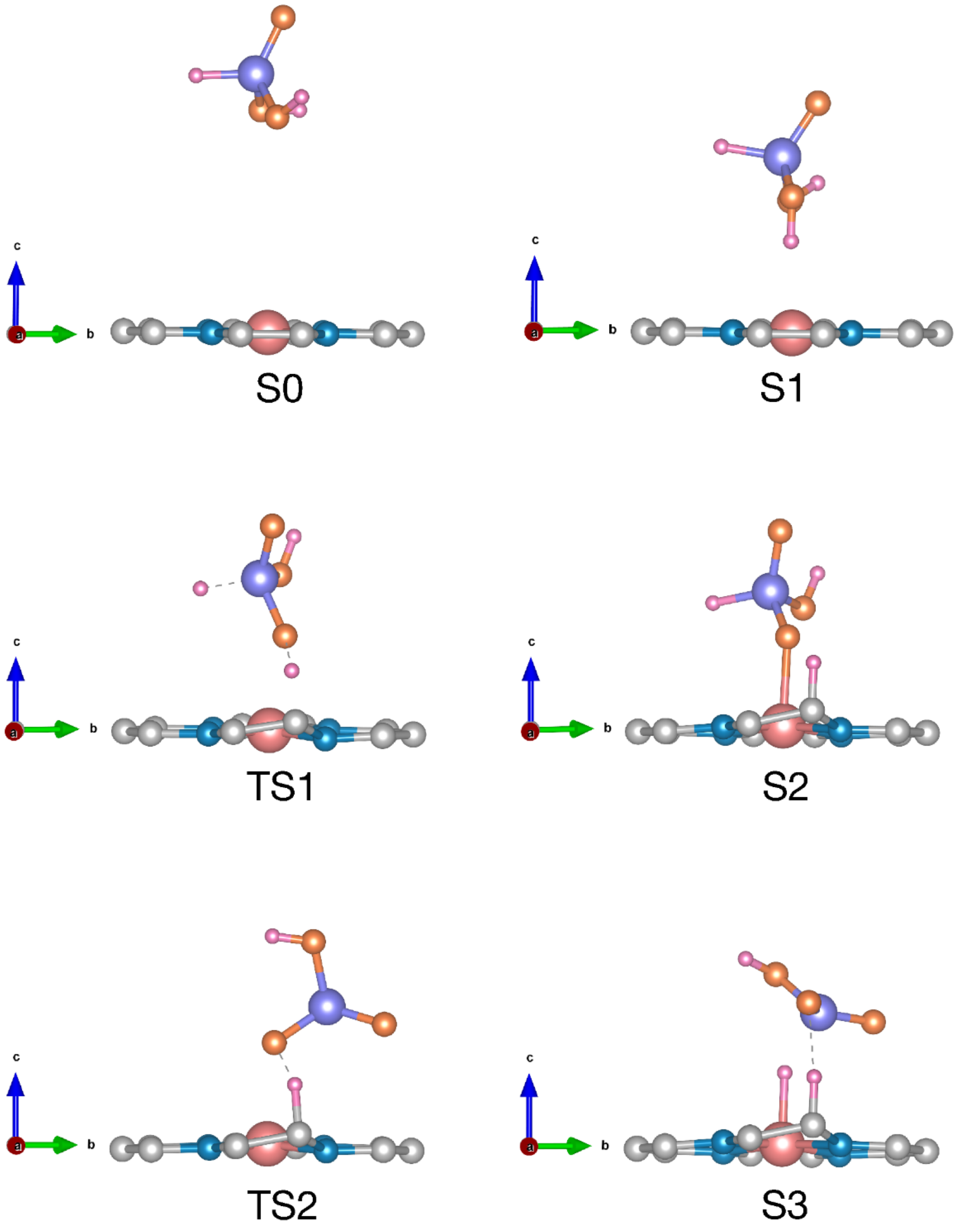

Supplementary Figure 29. Optimized structures of computational models in Rh-catalyzed H_3PO_3. blue, N; orange, O; gray, C; red, Rh; pink, H.

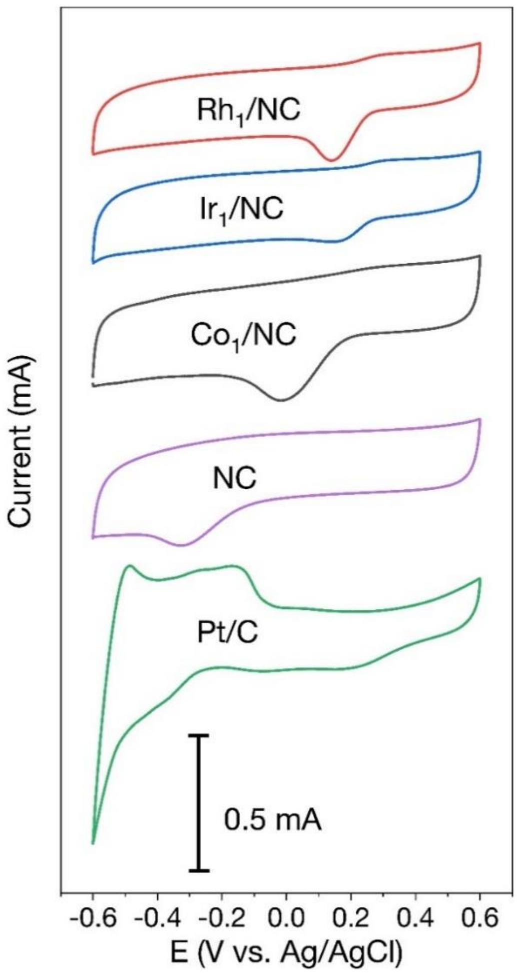

Supplementary Figure 30. CV curves of different catalysts in O_2-saturated 0.1 M acetate buffer + 0.1 M KCl (pH=4) with a scan rate of 100 mV s^{-1}.

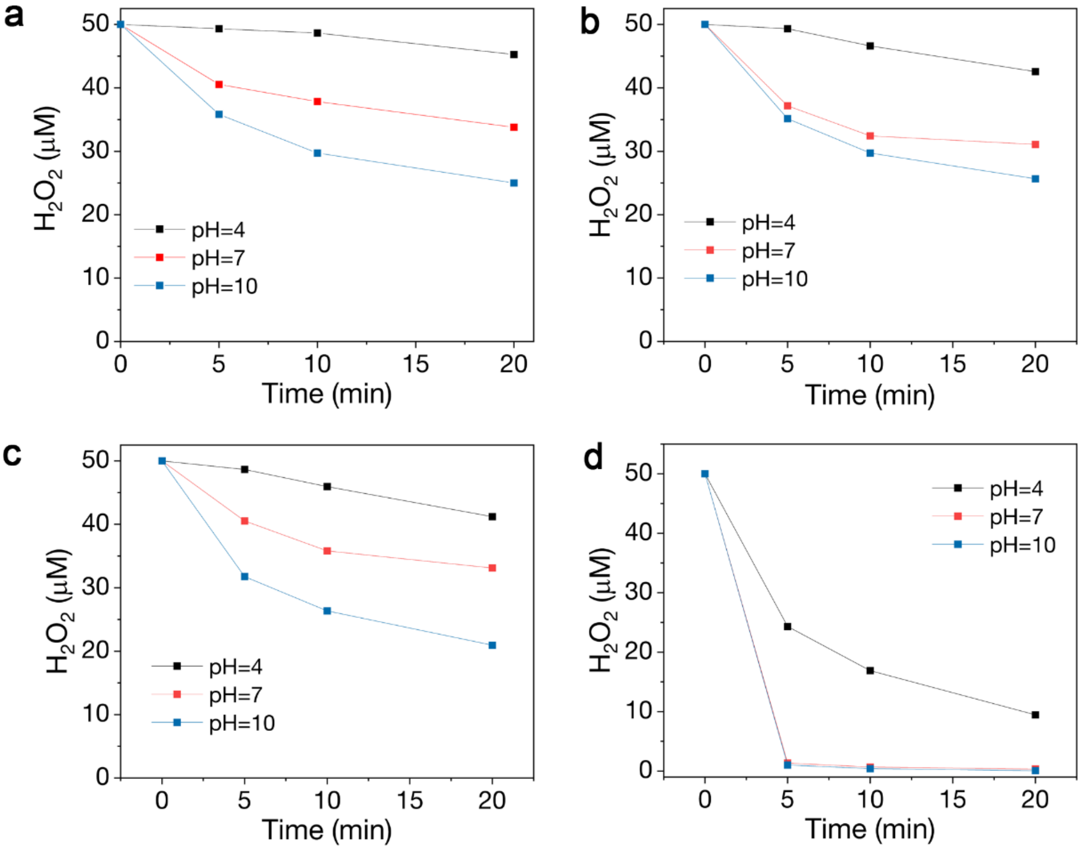

Supplementary Figure 31. H_2O_2 concentration variation with time in the presence of Co_1/NC (a), Rh_1/NC (b), Ir_1/NC (c), and Pt/C (d) (catalysts: 40 μg mL^{-1}).

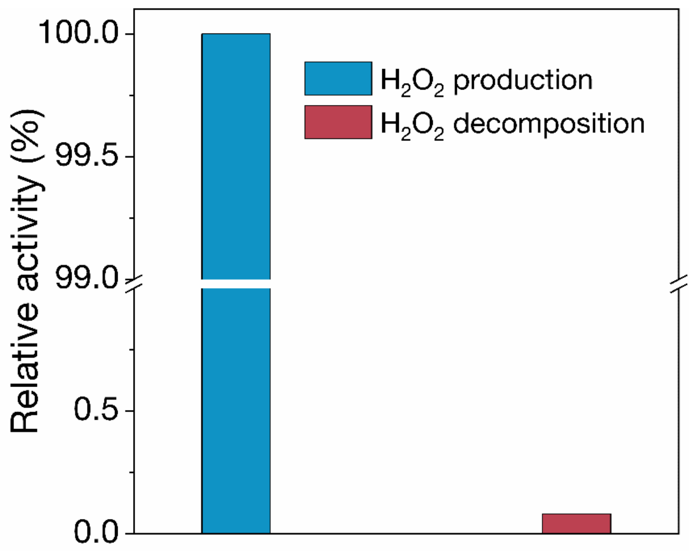

Supplementary Figure 32. The relative activity of the H_2O_2 production rate in the Rh_1/NC-catalyzed H_2PO_3 oxidation reaction and Rh_1/NC-catalyzed H_2O_2 decomposition rate in 0.1 M acetate buffer (pH=4).

The H_2O_2 production rate in the Rh_1/NC-catalyzed H_2PO_3 oxidation reaction is much higher than the decomposition rate of H_2O_2. This ensures that H_2O_2 can accumulate gradually.

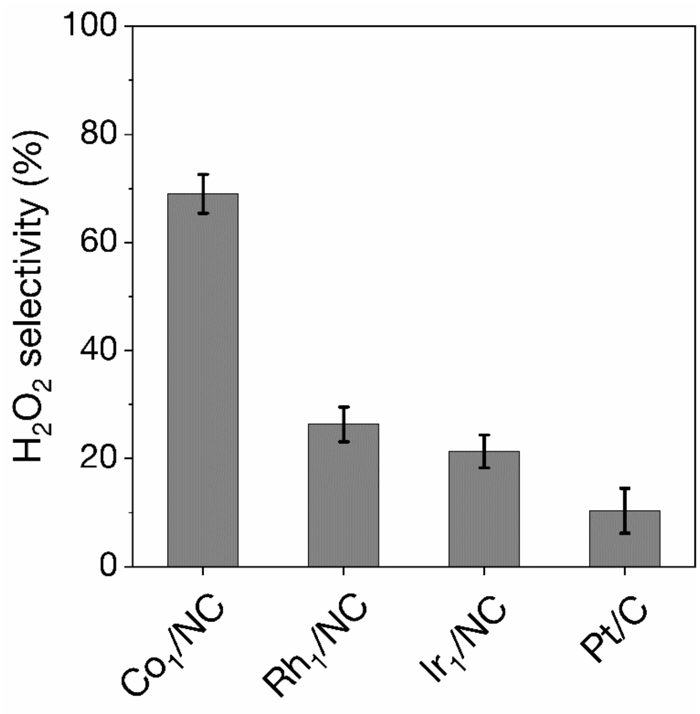

Supplementary Figure 33. H_2O_2 selectivity in NADH aerobic oxidation with different catalysts in 0.1 M PBS (pH=7). Data are presented as mean values (SD). (The error bar represents the standard deviation of 3 independent measurements).

Supplementary Table 1. Elemental composition of catalysts.				
	**C (wt%)**	**N (wt%)**	**O (wt%)**	**Metal (wt%)**
**Rh_1/NC**	86.2	10.1	2.2	0.89
**Ir_1/NC**	85.4	10.4	2.6	1.62
**Co_1/NC**	88.2	9.4	1.9	0.54

Supplementary Table 2. EXAFS fitting of Rh_1/NC				
**N**	**R (Å)**	**σ^2 (Å^2)**	**ΔE_0 (eV)**	**R factor**
3.96 ± 0.74	2.00	0.0062 ± 0.0025	0.058 ± 0.018	0.02

Supplementary Table 3. Comparing the performance of Rh_1/NC and electrocatalysts for O_2 reduction to H_2O_2.				
**Catalyst**	**pH**	**Productivity (mmol g_{cat}^{-1} h^{-1})**	**Selectivity (%)**	**Reference**
**Rh_1/NC**	**3**	**480**	**~100**	**This work**
Pt-Hg/C	1	/	96	*Nat. Mater.* **2013**, 12, 1137
O-CNTs	13	111.7	~90	*Nat. Catal.* **2018**, 1, 156.
F-mrGO	13	430	~100	*Nat. Catal.* **2018**, 1, 282
Pt_1/HSC	1	/	96	*Nat. Commun.* **2016**, 7, 10922
g-N-CNHs	13	/	63	*Chem* **2018**, 4, 106
Pt_1-CuS_x	1	546	~95	*Chem* **2019**, 5, 2099
Au-Pd	1	/	~95	*J. Am. Chem. Soc.* **2011**, 133, 19432
Au–Pt–Ni	13	/	~95	*Adv. Mater.* **2016**, 28, 9949−9955
Co_1-NG(O)	13	418	~80	*Nat. Mater.* **2020**, 19, 436
Co SAC	1	80	~90	*Chem* **2020**, 6, 658
Co-POC-O	13	/	~85	*Adv. Mater.* **2019**, 31, 1808173
Fe-CNT	14	~1600	~90	*Nat. Commun.* **2019**, 10, 3997
Co−N−C	13	193	~70	*J. Am. Chem. Soc.* **2019**, 141, 12372

**Supplementary Table 4. Comparing the performance of Rh_1/NC and photocatalysts for O_2 reduction to H_2O_2.**				
**Catalyst**	**Electron donor**	**Productivity (mmol g_{cat}^{-1} h^{-1})**	**Irradiation (nm)**	Reference
**Rh_1/NC**	**H_3PO_3**	**480**	**Without light**	**This work**
TAPD-$(Me)_2$ COF	EtOH	0.23	420-700	*J. Am. Chem. Soc.* **2020**, 142, 20107
PEI/C_3N_4	H_2O	0.21	1000 W/m^2	*ACS Catal.* **2020**, 10, 6, 3697
OPA/$Zr_{92.5}Ti_{7.5}$-MOF	Benzyl alcohol	3.9	>420	*J. Mater. Chem. A* **2020**, 8, 1904
$Au_{0.5}/TiO_2$	EtOH	0.53	>280	*ACS Catal.* **2012**, 2, 599
g-C_3N_4/AQ-COOH	2-propanol	0.36	>400	*Appl. Catal., B* **2018**, 229, 121
g-C_3N_4-CNTs	formic acid	0.036	>400	*Appl. Catal., B* **2018**, 224, 725

Because there is no unified method to measure the selectivity of photocatalytic O_2 reduction to H_2O_2, most articles do not provide the selectivity (%). It is worth noting that although much work has been devoted to the study of the photocatalytic production of H_2O_2, the productivity (mmol g_{cat}^{-1} h^{-1}) of photocatalysts is far lower than that of electrocatalysts (three orders of magnitude lower).

**Supplementary Table 5. Comparing the performance of Rh_1/NC and the catalysts for O_2 reduction to H_2O_2 with H_2 as protons and electrons donor.**				
**Catalyst**	**Pressure (MPa)**	**Productivity (mmol g_{cat}^{-1} h^{-1})**	**Selectivity (%)**	**Reference**
**Rh_1/NC**	**0.1**	**480**	**~100**	**This work**
2.5%Au-2.5%Pd/SiO_2	3.7	80	/	*Science* **2006**, 311, 362
2.5%Au-2.5%Pd/carbon	3.7	175	98	*Science* **2009**, 323, 1037
3wt%Pd-2 wt%Sn/TiO_2	3.7	61	96	*Science* **2016**, 351, 965
